# The influence of day 3 embryo cell number on the clinical pregnancy and live birth rates of day 5 single blastocyst transfer from frozen embryo transfer cycles

**DOI:** 10.1186/s12884-022-05337-z

**Published:** 2022-12-29

**Authors:** Jie Wang, Zhenyu Diao, Junshun Fang, Lihua Zhu, Zhipeng Xu, Fei Lin, Ningyuan Zhang, Linjun Chen

**Affiliations:** 1 Center for Reproductive Medicine and Obstetrics and Gynecology, Nanjing Drum Tower Hospital, Nanjing University Medical School, 210008 Nanjing, China; 2 Center for Molecular Reproductive Medicine, Nanjing University, 210008 Nanjing, China

**Keywords:** Embryo, Blastocyst, Frozen embryo transfer, Clinical pregnancy, Live birth

## Abstract

**Background:**

To evaluate the influence of day 3 embryo cell number on the clinical pregnancy and live birth rates of day 5 single blastocyst transfer in frozen embryo transfer (FET) cycles.

**Methods:**

Our retrospective study included 3761 day 5 single blastocyst FET cycles between January 2015 and December 2019. These FET cycles were divided into three groups according to the day 3 embryo cell number: 939 cycles in the < 8-cell group, 1224 cycles in the 8-cell group and 1598 cycles in the > 8-cell group. The clinical pregnancy and live birth rates were compared among the three groups.

**Results:**

The clinical pregnancy rate of day 5 single blastocyst transfer in FET cycles increased significantly as the day 3 embryo cell number increased (52.2%, 61.4% and 66.8%, *P* < 0.001). Similarly, the live birth rate increased significantly as the day 3 embryo cell number increased (42.7%, 49.8% and 54.9%, *P* < 0.001). The results of the subgroup analysis showed that the clinical pregnancy and live birth rates were not significantly different among the three groups when good-quality blastocysts were transferred. The clinical pregnancy and live birth rates increased significantly as the day 3 embryo cell number increased when fair- and poor-quality blastocysts were transferred.

**Conclusion:**

The day 3 embryo cell number needs to be considered when day 5 single blastocyst transfer is performed in FET cycles, especially when fair- and poor-quality blastocysts are used for transfer. The transfer of a day 5 single blastocyst derived from an embryo with faster development on day 3 may shorten the time to achieving a live birth.

## Background

In vitro fertilization (IVF)/intracytoplasmic sperm injection (ICSI) has become an effective method for the infertility treatment. Sperm-oocyte fusion results in a two-pronuclei zygote that develops into a cleavage-stage embryo on day 3 after insemination during the IVF/ICSI process. These embryos may contain different cell numbers, and their developmental potential differs according to these cell numbers. The Istanbul consensus is that the optimal developmental speed for a day 3 embryo is the achievement of the cleavage stage with 8 blastomeres on the third day after insemination [1].

The effects of the transfer of cleavage-stage embryos with different embryonic developmental speeds on clinical outcomes are controversial. Racowsky et al. [2] reported that for day 3 cleavage-stage embryo transfer, the highest live birth rate was obtained with the transfer of 8-cell embryos. Tomic et al. [3] found that an 8-cell embryo had the highest implantation potential for embryo transfer on day 3 after insemination. Additionally, > 10-cell good-quality embryos had comparable clinical pregnancy and live birth rates to those of 8-cell embryos [4]. Zhu et al. [5] reported that an embryo with ≥ 8 cells should be chosen for transfer in the first single embryo frozen embryo transfer (FET) cycle. Kroener et al. [6] found that > 9-cell embryos had a higher rate of aneuploidy than 6–9-cell embryos from biopsied day 3 embryos using comparative genomic hybridization. Therefore, they recommended 6–9-cell embryos as the first choice for transfer rather than > 9-cell embryos.

The studies mentioned above all focused on the effect of the transfer of cleavage-stage embryos with different cell numbers on the clinical outcomes. According to Li et al. [7], the implantation and clinical pregnancy rates of ≤ 6-cell embryos were significantly lower than those of 7–9-cell and > 9-cell embryos in fresh transfer cycles. However, the implantation and clinical pregnancy rates were not significantly different after single blastocyst transfer of ≤ 6-cell embryos compared with those of ≥ 7-cell FET cycles. Pons et al. [8] reported that > 11-cell embryos can give rise to a euploid blastocyst and a similar live birth rate to that of 8-cell embryos in preimplantation genetic testing (PGT) for aneuploidy cycles, although the implantation and live birth rates of euploid blastocysts from slow-cleaving (< 8-cell) embryos were significantly lower than those of 8-cell embryos in that study. In our study, a large sample of data was analysed to evaluate the influences of day 3 embryo cell number (< 8-cell, 8-cell and > 8-cell) on the clinical pregnancy and live birth rates of day 5 single blastocyst transfer in FET cycles.

## Materials and methods

### Patients

The present study included patients who underwent day 5 single blastocyst transfer in FET cycles and one transfer from each patient was analysed. The present study excluded patients with transferred blastocysts derived from donor oocytes or sperm, PGT or cryopreserved blastocysts on day 6. There were 3761 day 5 single blastocyst FET cycles between January 2015 and December 2019. These FET cycles were divided into three groups according to the day 3 embryo cell number: 939 cycles in the < 8-cell group, 1224 cycles in the 8-cell group and 1598 cycles in the > 8-cell group.

### Embryo culture and assessment

All oocytes were inseminated using IVF or ICSI methods according to semen parameters. A zygote with two pronuclei at 16–18 h after insemination was separately cultured in a single microdroplet. All embryos were cultured with G1-PLUS/G2-PLUS sequential medium (Vitrolife) in an incubator at 37 °C under conditions of 6% CO_2_, 5% O_2_ and 89% N_2_ with saturated humidity. Day 3 embryos were assessed according to the Istanbul consensus [1]. Blastocysts were assessed using the Gardner scoring system [9]. The present study divided the transferred blastocysts into three groups: good-quality (III-VIAA/III-VIAB/III-VIBA), fair-quality (III-VIBB), and poor-quality (III-VIAC/III-VIBC/III-VICA/III-VICB).

### Blastocyst vitrification and warming

Expanded blastocysts with artificial shrinkage by a laser were cryopreserved on day 5 or day 6 after insemination. The freezing sequence of blastocysts was based on the morphology of the vitrified blastocysts, namely, the blastocyst with the best morphology was prioritized to be vitrified and warmed. The vitrification and warming of the blastocysts were performed using vitrification and warming kits (KITAZATO). The criterion for blastocyst survival was re-expansion of the blastocyst after warming.

### FET cycle

Different protocols, including hormone replacement therapy, mild stimulation, gonadotropin (Gn) stimulation, and natural and modified natural cycles, were performed for endometrial preparation in FET cycles. Blastocyst warming was performed when the endometrium reached a sufficient thickness. A blastocyst with the best morphology was transferred into the uterus two hours after warming.

### Follow-up

The definitions of clinical pregnancy, live birth, ectopic pregnancy and miscarriage were based on the reported literature [10]. A gestational sac with a heartbeat detected by ultrasound between the fourth and sixth weeks after single blastocyst transfer indicated a clinical pregnancy. Live birth data were obtained from the parents after delivery.

### Statistical analyses

SPSS 22.0 software was used to analyse all the data. Continuous variables, including female age, female BMI, duration of infertility and endometrial thickness, are presented as the mean ± SD and were compared using an independent samples Kruskal-Wallis test. Categorical variables, including the pattern of infertility, endometrial preparation, pattern of insemination, evenness of the blastomeres and fragmentation of day 3 embryo, grade of transferred blastocysts, and the clinical pregnancy and live birth rates, are presented as percentages and were compared using the chi-square or Fisher’s exact test. A Bonferroni test was performed to compare rates between the three groups in pairs. Day 3 embryo cell number, female age, female BMI, duration of infertility, pattern of infertility, endometrial preparation, thickness of the endometrium, pattern of insemination, evenness of the blastomeres and fragmentation of day 3 embryo, grade of transferred blastocysts and previous transfer cycles were used as independent variables and clinical pregnancy or live birth rates were used as dependent variables. A logistic regression analysis was performed to analyse the correlation between the clinical pregnancy or live birth rates and these confounding factors mentioned above. *P* < 0.05 indicated statistical significance.

## Results

### Maternal and cycle characteristics

As shown in Table 1, no significant differences were observed in female age, female BMI, duration of infertility, endometrial preparation or endometrial thickness among the three groups. The proportion of IVF patients in the > 8-cell group was significantly higher than that in the < 8-cell or 8-cell group. The quality of transferred blastocysts increased significantly as the day 3 embryo cell number increased. The proportion of good-quality blastocyst transfer in the > 8-cell group was significantly higher than that in the < 8-cell or 8-cell group. The evenness of the blastomeres of day 3 embryo was significantly higher in the 8-cell group than that in the < 8-cell and > 8-cell groups, and the evenness of the blastomeres of day 3 embryo in the > 8-cell group was also significantly higher than that in the < 8-cell group. The fragmentation of day 3 embryo was significantly decreased as day 3 embryo cell number increased. The proportion of previous transfer cycles did not differ significantly between the 8-cell group and the > 8-cell group, but it was significantly lower than that of the < 8-cell group. Thus, the transferred blastocysts derived from the faster developing embryos may shorten the time to pregnancy or live birth.

**Table 1:** Maternal and cycle characteristics among the different groups

	**< 8-cell**	**8-cell**	**> 8-cell**	***P*-value**
Number of transfer cycles	939	1224	1598	—
Female age (years)	31.5±4.4	31.4±4.4	31.1±4.5	0.064
Female BMI (kg/m^2^)	22.7±3.3	22.4±3.3	22.7±3.3	0.084
Duration of infertility (years)	3.7±2.8	3.6±2.7	3.5±2.7	0.317
Pattern of infertility				
Primary	473(50.4)^a^	558(45.6)	791(49.5)	0.047
Secondary	466(49.6)	666(54.4)	807(50.5)	—
Endometrial preparation				
HRT cycles	774(82.4)	1022(83.5)	1345(84.2)	0.518
Mild stimulation cycles	31(3.3)	26(2.1)	56(3.5)	0.078
Gn stimulation cycles	24(2.6)	29(2.4)	43(2.7)	0.865
Natural cycles	6(0.6)	15(1.2)	17(1.1)	0.378
Modified natural cycles	104(11.1)	132(10.8)	137(8.6)	0.055
Thickness of endometrium (mm)	9.6±1.6	9.5±1.6	9.6±1.7	0.359
Pattern of insemination				
IVF	706(75.2)^b^	926(75.7)^b^	1370(85.7)^c^	<0.001
ICSI	233(24.8)	298(24.3)	228(14.3)	—
Evenness of the blastomeres of day 3 embryo				
Even	654(69.6)^b^	1001(81.8)^c^	1191(74.5)^d^	<0.001
Uneven	285(30.4)	223(18.2)	407(25.5)	—
Fragmentation of day 3 embryo				
<10%	471(50.2)^b^	780(63.7)^c^	1183(74.0)^d^	<0.001
10-25%	441(47.0)^b^	439(35.9)^c^	411(25.7)^d^	<0.001
>25%	27(2.9)^b^	5(0.4)^c^	4(0.3)^c^	<0.001
Grade of transferred blastocysts				
Good quality	143(15.2)^b^	379(31.0)^c^	598(37.4)^d^	<0.001
Fair quality	487(51.9)^b^	629(51.4)^b^	746(46.7)^c^	0.012
Poor quality	309(32.9)^b^	216(17.6)^c^	254(15.9)^c^	<0.001
Previous transfer cycles				
0	336(35.8)^b^	561(45.8)^c^	739(46.2)^c^	<0.001
1	428(45.6)^b^	468(38.2)^c^	632(39.5)^c^	0.001
2	115(12.2)	130(10.6)	162(10.1)	0.250
≥3	60(6.4)^b^	65(5.3)^b,c^	65(4.1)^c^	0.031

^a^values in parenthesis are expressed in percentage

different letters b, c and d represent significant differences (Bonferroni test)

### Grade of transferred blastocysts

As shown in Table 2, the expansion grade was significantly higher as day 3 embryo cell number increased. The proportion of good-quality blastocysts increased significantly as day 3 embryo cell number increased. The proportion of poor-quality blastocysts decreased significantly as day 3 embryo cell number increased.

**Table 2:** Grade of transferred blastocysts among the different groups

	**< 8-cell**	**8-cell**	**> 8-cell**	***P*-value**
Total transferred blastocysts	939	1224	1598	—
Expansion grade				
III	90(9.6)^a^^,b^	81(6.6)^c^	48(3.0)^d^	<0.001
IV	728(77.5)^b,c^	978(79.9)^b^	1216(76.1)^c^	0.054
V	101(10.8)^b^	135(11.0)^b^	226(14.1)^c^	0.012
VI	20(2.1)^b^	30(2.5)^b^	108(6.8)^c^	<0.001
Good quality				
IIIAA/IIIAB/IIIBA	2(0.2)	5(0.4)	3(0.2)	0.567
IVAA/IVAB/IVBA	120(12.8)^b^	309(25.2)^c^	427(26.7)^c^	<0.001
VAA/VAB/VBA	18(1.9)^b^	47(3.8)^c^	109(6.8)^d^	<0.001
VIAA/VIAB/VIBA	3(0.3)^b^	18(1.5)^c^	59(3.7)^d^	<0.001
Fair quality				
IIIBB	36(3.8)^b^	33(2.7)^b^	14(0.9)^c^	<0.001
IVBB	383(40.8)	511(41.7)	599(37.5)	0.052
VBB	57(6.1)	75(6.1)	95(5.9)	0.980
VIBB	11(1.2)^b,c^	10(0.8)^b^	38(2.4)^c^	0.002
Poor quality				
IIIAC/IIIBC/IIICA/IIICB	52(5.5)^b^	43(3.5)^b^	31(1.9)^c^	<0.001
IVAC/IVBC/IVCA/IVCB	225(24.0)^b^	158(12.9)^c^	190(11.9)^c^	<0.001
VAC/VBC/VCA/VCB	26(2.8)^b^	13(1.1)^c^	22(1.4)^c^	0.007
VIAC/VIBC/VICA/VICB	6(0.6)	2(0.2)	11(0.7)	0.097

^a^values in parenthesis are expressed in percentage

different letters b, c and d represent significant differences (Bonferroni test)

### Clinical outcomes

As shown in Table 3, the clinical pregnancy and live birth rates increased significantly as the day 3 embryo cell number increased. There were no significant differences in the rates of miscarriage among the three groups. The rate of termination of pregnancy in the > 8-cell group was significantly higher than that in the < 8-cell group.

**Table 3:** Clinical outcomes among the different groups

	< 8-cell	8-cell	> 8-cell	*P*-value
Number of transfer cycles	939	1224	1598	—
Number of clinical pregnancy cycles	490 (52.2)^a,b^	751(61.4)^c^	1068(66.8)^d^	< 0.001
Number of ectopic pregnancy cycles	8 (1.6)	9 (1.2)	6 (0.6)	0.102
Number of miscarriage cycles	79 (16.1)	126 (16.8)	164 (15.4)	0.711
Number of termination of pregnancy cycles	1 (0.2)^b^	5 (0.7)^b,c^	19 (1.8)^c^	0.008
Number of live birth cycles (per transfer cycle)	401 (42.7)^b^	609 (49.8)^c^	878 (54.9)^d^	< 0.001

^a^values in parenthesis are expressed in percentage

different letters b, c and d represent significant differences (Bonferroni test)

### The effects of the confounding factors on the clinical outcomes

The results of logistic regression analysis of clinical pregnancy and live birth rates after adjustments for confounding factors are shown in Table 4. Day 3 embryo cell number was a significant factor affecting the rates of clinical pregnancy (adjusted Odds Ratio (aOR) 1.057, 95% confidence interval (CI) 1.030–1.086) and live birth (aOR 1.054, 95% CI 1.027–1.081) by logistic regression analysis. Moreover, female age, endometrial thickness, transferred blastocyst grade and the previous transfer cycles also significantly affected the clinical pregnancy and live birth rates.

**Table 4:** Results of the logistic regression analysis of clinical pregnancy and live birth rates after adjustments for confounding factors according to the maternal and cycle characteristics

	**Clinical pregnancy rate**			**Live birth rate**		
	**aOR**	**95% CI**	***P*-value**	**aOR**	**95% CI**	***P*-value**
Female age	0.951	0.934-0.967	<0.001	0.932	0.916-0.948	<0.001
Female BMI	0.997	0.976-1.018	0.761	0.986	0.966-1.006	0.179
Duration of infertility	0.987	0.962-1.014	0.345	0.989	0.963-1.015	0.395
Pattern of infertility	1.005	0.867-1.166	0.944	1.027	0.888-1.187	0.719
Endometrial preparation	0.971	0.919-1.025	0.283	1.016	0.963-1.072	0.553
Thickness of endometrium	1.123	1.075-1.174	<0.001	1.099	1.053-1.146	<0.001
Pattern of insemination	0.880	0.741-1.046	0.148	0.912	0.770-1.079	0.282
Day 3 embryo cell number	1.057	1.030-1.086	<0.001	1.054	1.027-1.081	<0.001
Evenness of the blastomeres of day 3 embryo	0.887	0.757-1.040	0.140	0.890	0.761-1.041	0.144
Fragmentation of day 3 embryo	0.878	0.763-1.011	0.071	0.981	0.853-1.128	0.785
Grade of transferred blastocysts	1.357	1.225-1.503	<0.001	1.381	1.248-1.527	<0.001
Previous transfer cycles	0.914	0.850-0.983	0.015	0.900	0.835-0.969	0.005

### Effect of the transferred blastocyst grade on the clinical outcomes

A subgroup analysis of the effect of the transferred blastocyst grade on the clinical pregnancy and live birth rates was shown in Table 5. For good-quality transferred blastocysts, the clinical pregnancy and live birth rates were not significantly different among the three groups. The clinical pregnancy and live birth rates in the fair-quality and poor-quality subgroups of transferred blastocysts increased significantly as the day 3 embryo cell number increased.

**Table 5:** The effect of transferred blastocyst grade on the rates of clinical pregnancy and live birth

**Blastocyst grade **		**< 8-cell**	**8-cell**	**> 8-cell**	***P*-value**
Good	Number of transfer cycles	143	379	598	—
	Number of clinical pregnancy	97 (67.8)^a^	254 (67.0)	421 (70.4)	0.507
	Number of live birth (per transfer cycle)	82 (57.3)	218 (57.5)	359 (60.0)	0.688
Fair	Number of transfer cycles	487	629	746	—
	Number of clinical pregnancy	270 (55.4)^b^	381 (60.6)^b^	509 (68.2)^c^	<0.001
	Number of live birth (per transfer cycle)	216 (44.4)^b^	314 (49.9)^b,c^	409 (54.8)^c^	0.001
Poor	Number of transfer cycles	309	216	254	—
	Number of clinical pregnancy	123 (39.8)^b^	116 (53.7)^c^	138 (54.3)^c^	0.001
	Number of live birth (per transfer cycle)	103 (33.3)^b^	77 (35.6)^b,c^	110 (43.3)^c^	0.045

^a^values in parenthesis are expressed in percentage

different letters b and c represent significant differences (Bonferroni test)

## Discussion

The present study suggested that the clinical and live birth rates of day 5 single blastocyst transfer in FET cycles increased significantly as the day 3 embryo cell number increased. However, the miscarriage rate of day 5 single blastocyst transfer in FET cycles was not significantly affected by the day 3 embryo cell number.

The transfer of cleavage-stage embryos with different numbers of blastomeres due to inconsistent developmental speeds during the process of in vitro embryo culture affects clinical outcomes [3, 4, 6, 11]. The extended culture of embryos after IVF to produce blastocysts for single blastocyst transfer has become an effective method of assisted pregnancy to reduce the rate of multiple pregnancies without reducing the clinical pregnancy rate compared with double cleavage-stage embryo transfer [12, 13]. Several factors, including maternal age, fertilization mode and in vitro culture conditions, can affect the blastocyst formation rate of human embryos cultured in vitro [14–16]. In addition, the day 3 embryo cell number, a characteristic specific to the embryo, also significantly affects the blastocyst formation rate of human embryos cultured in vitro. It was found that the blastocyst formation rate was positively associated with day 3 embryo cell number and, in particular, good-quality blastocysts [8, 14]. The present study showed that the quality of the transferred blastocyst increased significantly as the day 3 embryo cell number increased, which was consistent with the studies mentioned above. A higher proportion of good-quality blastocysts in the > 8-cell group may also be related to the higher proportion of IVF patients in this group, as embryos derived from conventional IVF may have a higher rate of blastocyst formation than those derived from ICSI [17]. Guo et al. [18] reported that blastocyst quality exerted significant effects on the live birth rate. The results of our retrospective study were similar, and the clinical outcomes also increased significantly as the day 3 embryo cell number increased, that is, transferred blastocysts derived from embryos with fast development can lead to higher clinical pregnancy and live birth rates. Moreover, day 3 embryo cell number did not significantly affect the miscarriage rate. However, the rate of termination of pregnancy due to foetal anomalies and pregnancy complications in the > 8-cell group was significantly higher than that in the < 8-cell group. Although the > 8-cell group had a higher pregnancy termination rate, it did not significantly reduce the live birth rate. The fact that faster developing embryos may lead to significantly increased rates of termination of pregnancy requires further research.

The morphology of the transferred blastocyst also affects clinical outcomes. Zhao et al. [19] found that the live birth rate of the ≤ 6-cell group was significantly lower than that of the 8-cell group in single blastocyst FET cycles when fair-quality (IVBB) blastocysts were transferred. The miscarriage rate of the former group was significantly higher than that of the latter group. The clinical pregnancy, live birth and early miscarriage rates were not significantly different between the two groups when high-quality (more than IVBB) blastocysts were transferred. When we conducted a subgroup analysis of the transferred blastocysts, we found that the clinical pregnancy and live birth rates of the good-quality blastocysts increased as the day 3 embryo cell number increased, but there was no significant difference between groups; however, the clinical pregnancy and live birth rates of fair-quality or poor-quality blastocysts increased significantly as the day 3 embryo cell number increased. The results of our study were consistent with the reports mentioned above. Ozgur et al. [20] found that the live birth rate of a single best unknown-ploidy blastocyst was equivalent to that of a single best euploid blastocyst from patients under 35 years old who underwent FET cycles, which may be related to the positive correlation between the euploid rate of the blastocyst and the morphology of the blastocyst [21]. Therefore, the clinical outcomes of blastocysts derived from faster-developing cleavage-stage embryos were superior to those of blastocysts derived from slower-developing cleavage-stage embryos. This result may be related to the tendency for faster-developing cleavage-stage embryos to produce a higher rate of high-quality euploid blastocysts.

The euploid rate of the transferred embryos or blastocysts was positively correlated with the clinical pregnancy and live birth rates [22, 23]. In addition to the positive correlation between the rate of blastocyst formation and day 3 embryo cell number, the euploid rate of blastocysts was also positively associated with day 3 embryo cell number [8]. Wu et al. [24] reported that a transferred blastocyst derived from a low day 3 embryo cell number (≤ 5 cells) significantly decreased the live birth rate. The poor clinical outcomes of the transferred blastocysts derived from cleavage-stage embryos with a low day 3 embryo cell number may be related to the lower euploid rate of these blastocysts. In the present study, patients with the transferred blastocysts derived from the low day 3 embryo cell number also had poor clinical outcomes, although these blastocysts did not undergo PGT. However, Pons et al. [8] reported that the live birth rate of euploid blastocysts derived from slow-cleaving embryos was significantly lower than that of euploid blastocysts derived from 8-cell embryos, while the live birth rate of euploid blastocysts derived from fast-cleaving embryos was equivalent to that of 8-cell embryos. There may be other intrinsic embryonic factors (the differential expression of RNA and epigenetic modification) affecting the clinical outcomes of embryos with fast development compared to those with slow development.

The limitations of our study were its retrospective nature and use of data from our own reproductive centre. Although all patients underwent one transfer during this period in our retrospective study, some patients underwent previous transfer cycles. Therefore, the inclusion of these patients may be a source of selection bias, which was another limitation of our study. The strengths of the present study were its use of a large sample of data to demonstrate the association between the clinical pregnancy and live birth rates of day 5 single blastocyst transfer in FET cycles and day 3 embryo cell number. Another strength of our study was its inclusion of day 5 single blastocyst transfer data from FET cycles, and day 6 single blastocyst transfer cycles were excluded to avoid the selection bias associated with the in vitro culture time on the clinical outcomes of single blastocyst transfer.

## Conclusion

The day 3 embryo cell number needs to be considered when day 5 single blastocyst transfer is performed in FET cycles, especially when fair- and poor-quality blastocysts were performed for transfer. The transfer of a day 5 single blastocyst derived from an embryo with faster development on day 3 may shorten the time to achieving a live birth.

## Data Availability

The datasets used and/or analyzed during the current study are available from. the corresponding author on request.

## Abbreviations

FET: Frozen embryo transfer
IVF: In vitro fertilization
ICSI: Intracytoplasmic sperm injection
PGT: Preimplantation genetic testing
Gn: Gonadotropin
aOR: Adjusted Odds Ratio
CI: Confidence interval
